# Supplementary material for: White-handed gibbons discriminate context-specific song compositions
Source: PeerJ. 2020 Aug 3;8:e9477. doi: 10.7717/peerj.9477 (PMC7409784; doi:10.7717/peerj.9477)
Supplement: Supplemental Information 1 — § Nduet songs = 3. Groups A, W and T did not produce any ‘sharp wow’ note in their duet songs. ** means ± SD [file peerj-08-9477-s001.docx]

**Table S1.** Overview of playback stimuli characteristics (N_duet songs_=6, N_leopard songs_=6)

| Variables** | Duet stimuli | Predator stimuli |
| --- | --- | --- |
| Introductory ‘hoo’ note series (s) | 6.4 ± 8.1 | 18.8 ± 12.1 |
| Number of introductory ‘hoo’ notes | 6.8 ± 3.7 | 40.5 ± 823.6 |
| Song duration (s) | 1124.9 ± 217.1 | 2632.7 ± 816.5 |
| Latency to first great call (s) | 105.5 ± 20.0 | 1094.5 ± 650.5 |
| ^§^Latency to first ‘sharp wow’ (s) | 115.4 ± 66.1 | 354.0 ± 182.2 |
| ^§^Number of ‘sharp wow’ notes | 5.5 ± 7.5 | 424.0 ± 239.9 |

^§^ N_duet songs_ = 3. Groups A, W and T did not produce any ‘sharp wow’ note in their duet songs. ** means ± SD
