# Supplementary material for: White-handed gibbons discriminate context-specific song compositions
Source: PeerJ. 2020 Aug 3;8:e9477. doi: 10.7717/peerj.9477 (PMC7409784; doi:10.7717/peerj.9477)
Supplement: Supplemental Information 2 [file peerj-08-9477-s002.docx]

Table S2. Playback experimental design

| Trial | Tested group | Stimuli group | Song type |
| --- | --- | --- | --- |
| 1 | W | S | Predator song |
| 2 | W | S | Duet |
| 3 | R | M | Duet |
| 4 | H | A | Predator song |
| 5 | B | T | Duet |
| 6 | M | N | Duet |
| 7 | M | N | Predator song |
| 8 | R | M | Predator song |
| 9 | H | A | Duet |
| 10 | N | W | Predator song |
| 11 | B | T | Predator song |
| 12 | N | W | Duet |
