# Supplementary material for: White-handed gibbons discriminate context-specific song compositions
Source: PeerJ. 2020 Aug 3;8:e9477. doi: 10.7717/peerj.9477 (PMC7409784; doi:10.7717/peerj.9477)
Supplement: Supplemental Information 3 — #Negative values can emerge because all vocal responses to duet playbacks temporally overlapped the stimulus. ** means ± SD. [file peerj-08-9477-s003.docx]

Table S3. Overview of group vocal responses to playback treatments

| Variables** | Duet playback  (N = 5 responding groups) | Predator playback  (N = 1 responding group) |
| --- | --- | --- |
| Latency to reply to stimulus (s) | 678.1 ± 70.0 | 3238.4 |
| Difference between stimulus offset and response onset (s) | -358.5 ± 75.9^#^ | 580.9 |
| Song duration (s) | 1006.8 ± 122.3 | 1073.5 |
| Latency to first great call (s) | 99.0 ± 41.1 | 819.4 |
| Latency to first ‘sharp wow’ (s) | 90.8 ± 35.9 | 193.1 |

^#^Negative values can emerge because all vocal responses to duet playbacks temporally overlapped the stimulus. ** means ± SD.
