# Supplementary material for: White-handed gibbons discriminate context-specific song compositions
Source: PeerJ. 2020 Aug 3;8:e9477. doi: 10.7717/peerj.9477 (PMC7409784; doi:10.7717/peerj.9477)
Supplement: Supplemental Information 4 — ** means ± SD. [file peerj-08-9477-s004.docx]

Table S4. Overview of other groups’ responses to playback treatments

| Variables** | Duet playback  (N = 8 responding groups) | Predator playback  (N = 2 responding groups) |
| --- | --- | --- |
| Song duration (s) | 768.6 ± 175.8 | 1425.9 ± 404.5 |
| Latency to first great call (s) | 99.0 ± 37.6 | 493.7 ± 196.9 |

** means ± SD.
