# Supplementary material for: White-handed gibbons discriminate context-specific song compositions
Source: PeerJ. 2020 Aug 3;8:e9477. doi: 10.7717/peerj.9477 (PMC7409784; doi:10.7717/peerj.9477)
Supplement: Supplemental Information 5 — ¤ As there are ties present in the data, Dunn’s test with Benjamini & Hochberg corrections was used to perform multiple pairwise comparisons between song types on the number of ‘sharp wow’ notes so as to correct z-quantiles for ties. (*P < 0.05). Predator songs were found to be introduced by a longer ‘hoo’ note series, that also contains more ‘hoo’ notes than spontaneous duet songs and duet playback responses (mean ‘hoo’ duration: spontaneous duet: 8.2 ± 3.5 s; playback duet: 4.7 ± 2.7 s; leopard song: 23.4 ± 6.7 s; mean ‘hoo’notes number: spontaneous duet: 11.0 ± 4.5; playback duet: 7.4 ± 2.7; leopard song: 48.8 ± 11.4). Furthermore, predator songs were found to be longer in duration with a delayed first great call production compared to spontaneous duet songs and duet playback responses (mean song duration: spontaneous duet: 794.5 ± 340.1 s; playback duet: 1,006.8 ± 122.3 s; leopard song: 2,396.4 ± 775.8 s; mean latency to first great call: spontaneous duet: 104.8 ± 37.6 s; playback duet: 99.0 ± 41.1 s; leopard song: 816.4 ± 368.0 s). Additionally, differences emerged when analysing the production latency of the first ‘sharp wow’ and in the number of ‘sharp wow’ notes produced, with predator songs containing more ‘sharp wow’ notes with a delayed production (mean latency to first ‘sharp wow’: spontaneous duet: 78.1 ± 31.1 s; playback duet: 90.8 ± 35.9 s; leopard song: 370.5 ± 183.2 s; mean ‘sharp wow’ notes number: spontaneous duet: 11.5 ± 7.1; playback duet: 5.6 ± 6.0; leopard song: 362.2 ± 233.9). [file peerj-08-9477-s005.docx]

Table S5. Comparison of songs given in response to duet playbacks with spontaneous duets and clouded leopard songs given by the same groups (Pairwise comparisons using Wilcoxon rank sum test, with Benjamini & Hochberg corrections).

| **Context 1** | **Context 2** | ***P* value** |
| --- | --- | --- |
| ***Introductory ‘hoo’ series duration (s)*** | | |
| Playback duet response | Spontaneous duet  Leopard song | 0.151  <0.05* |
| Spontaneous duet | Leopard song | <0.05* |
| ***Number of introductory ‘hoo’ notes*** | | |
| Playback duet response | Spontaneous duet  Leopard song | 0.22  <0.05* |
| Spontaneous duet | Leopard song | <0.05* |
| ***Song duration (s)*** | | |
| Playback duet response | Spontaneous duet  Leopard song | 0.222  <0.05* |
| Spontaneous duet | Leopard song | <0.05* |
| ***Latency to 1^st^ great call (s)*** | | |
| Playback duet response | Spontaneous duet  Leopard song | 0.690  <0.05* |
| Spontaneous duet | Leopard song | <0.05* |
| ***Latency to 1^st^ ‘sharp wow’ (s)*** | | |
| Playback duet response | Spontaneous duet  Leopard song | 0.905  <0.05* |
| Spontaneous duet | Leopard song | <0.05* |
| ***Number of ‘sharp wow’* ^¤^** | | |
| Playback duet response | Spontaneous duet  Leopard song | 0.524  <0.01* |
| Spontaneous duet | Leopard song | <0.05* |

^¤^ As there are ties present in the data, Dunn’s test with Benjamini & Hochberg corrections was used to perform multiple pairwise comparisons between song types on the number of ‘sharp wow’ notes so as to correct z-quantiles for ties. (* P<0.05).
