# Supplementary material for: White-handed gibbons discriminate context-specific song compositions
Source: PeerJ. 2020 Aug 3;8:e9477. doi: 10.7717/peerj.9477 (PMC7409784; doi:10.7717/peerj.9477)
Supplement: Supplemental Information 6 — (*P < 0.05). Leopard songs were found to contain more ‘hoo’ notes and less ‘leaning wa’ than spontaneous duets and playback duet songs, with no differences between spontaneous duets and playback duet songs (‘hoo’: spontaneous duet: 1.2 ± 1.3; playback duet: 1.2 ± 1.5; leopard song: 6.1 ± 2.5; ‘leaning wa’: spontaneous duet: 1.0 ± 1.6; playback duet: 1.0 ± 1.3; leopard song: 0.2 ± 0.6). However, songs in response to duet playbacks contained more ‘wa-oo’ notes than spontaneous duet songs and predator songs, with spontaneous duet songs containing also more ‘wa-oo’ notes than predator songs (‘wa-oo’: spontaneous duet: 1.8 ± 2.0; playback duet: 5.0 ± 2.3; leopard song: 0.1 ± 0.3). [file peerj-08-9477-s006.docx]

Table S6. Comparison of the first ten notes produced across singing contexts (duet playback responses, spontaneous duets and clouded leopard songs given by the same five groups, N=15) (Pairwise comparisons using Chi-squared post-hoc tests, with Benjamini & Hochberg corrections, n=300 notes).

| **Context 1** | **Context 2** | *P* value |
| --- | --- | --- |
| ***‘hoo’ notes*** | | |
| Playback duet response | Spontaneous duet  Leopard song | 1  <0.001* |
| Spontaneous duet | Leopard song | <0.001* |
| ***‘leaning wa’ notes*** | | |
| Playback duet response | Spontaneous duet  Leopard song | 1  <0.05* |
| Spontaneous duet | Leopard song | <0.05* |
| ***‘wa-oo’ notes*** | | |
| Playback duet response | Spontaneous duet  Leopard song | <0.001*  <0.001* |
| Spontaneous duet | Leopard song | <0.001* |

(* P<0.05).
